# Remarkable preservation of terpenoids and record of volatile signalling in plant-animal interactions from Miocene amber

**DOI:** 10.1038/s41598-017-09385-w

**Published:** 2017-09-08

**Authors:** Suryendu Dutta, Rakesh C. Mehrotra, Swagata Paul, R. P. Tiwari, Sharmila Bhattacharya, Gaurav Srivastava, V. Z. Ralte, C. Zoramthara

**Affiliations:** 1 Department of Earth Sciences, Indian Institute of Technology Bombay, Mumbai, 400076 India; 2 Birbal Sahni Institute of Palaeosciences, Lucknow, 226007 India; 3 Department of Geology, Mizoram University, Aizawl, 796004 India; 4 Dr. H. S. Gour Vishwavidyalaya, Sagar, 470003 Madhya Pradesh India

## Abstract

Plants produce and release a large array of volatile organic compounds that play many ecological functions. These volatile plant metabolites serve as pollinator attractants, herbivore and pathogen repellents and protect plants from abiotic stresses. To date, the geological evolution of these organic compounds remains unknown. The preservation potential of these metabolites in the fossil record is very poor due to their low boiling points. Here we report a series of volatile sesquiterpenoids, including δ-elemene, α-copaene, β-elemene, β-caryophyllene, α-humulene, germacrene D, δ-cadiene and spathunenol, from early Miocene (~17 million year) amber from eastern India. The survival of these unaltered bioterpenoids can be attributed to the existence of extraordinary taphonomic conditions conducive to the preservation of volatile biomolecules through deep time. Furthermore, the occurrence of these volatiles in the early Miocene amber suggests that the plants from this period had evolved metabolic pathways to synthesize these organic molecules to play an active role in forest ecology, especially in plant-animal interactions.

## Introduction

Plants synthesize and release a vast array of volatiles organic compounds that are helpful in their interactions with their immediate environment^1, 2^. These metabolites protect plant cells from biotic and abiotic stresses^3^. The volatile compounds can repel predators or defend the plant by attracting natural enemies of herbivores^4, 5^. The modern genera of higher plants produce up to several hundred volatile terpenoids, but their geological evolution remains unknown. Natural products are readily degraded during diagenesis. This includes the transformation of bioterpenoids into geoterpenoid during fossilization, some of which may maintain structural resemblance to their terpenoid precursors^6^. However, it is often difficult to elucidate the biological precursors of many geoterpenoids or the biosynthetic mechanisms that produce them. Additionally, the volatile terpenoids are susceptible to diagenesis because of their low boiling points. Here we report a group of biogenic volatile sesquiterpenes from Miocene amber from northeastern India. The present study suggests that plants had evolved volatile terpene-based compounds in deep time to participate in ecological interactions.

## Results and Discussion

**Figure 1:**
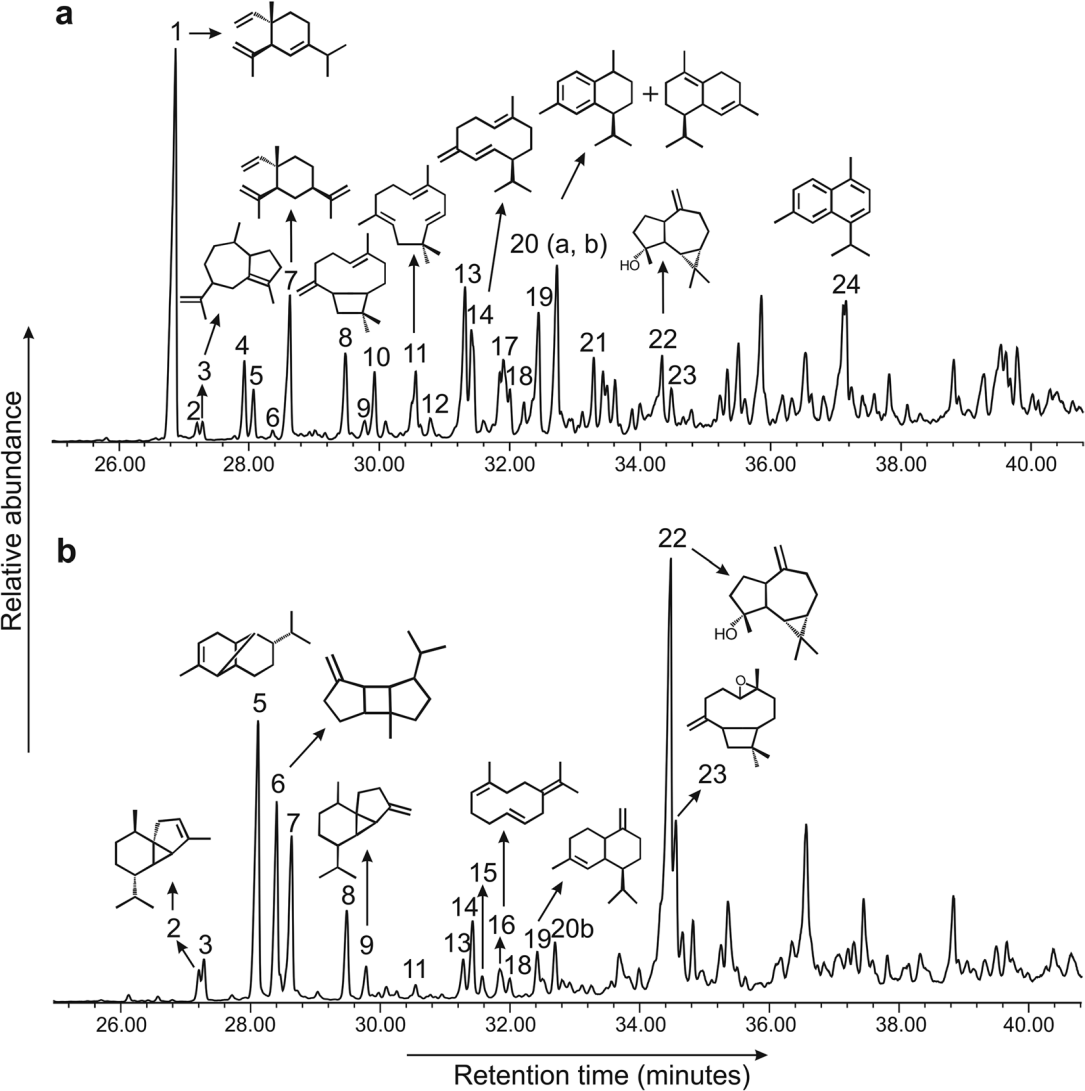
Gas chromatography-mass spectrometry traces (reconstructed) of (**a**) total ion current of the total extract of the Miocene amber from Bhuban Formation, Assam Basin, northeastern India, (**b**) total ion current of the total extract of the of the extant dammar resin of *Shorea robusta*. The numbers indicate sequiterpenoids present in the studied samples: 1, δ-elemene; 2, α-cubebene; 3, aciphyllene; 4, unknown sesquiterpenoid; 5, α-copaene; 6, β-bourbonene; 7, β-elemene; 8, β-caryophyllene; 9, β-cubebene; 10, unknown sesquiterpenoid; 11, α-humulene; 12, octahydro naphthalene; 13, muurolene isomer; 14, germacrene D; 15, viridoflorene; 16, germacrene B; 17, octahydro naphthalene; 18, α-muurolene; 19, γ-cadinene; 20, calamenene (**a**) & δ-cadinene (**b**); 21, α-calacorene; 22, spathulenol; 23, caryphyllene oxide; 24, cadalene.

**Figure 2:**
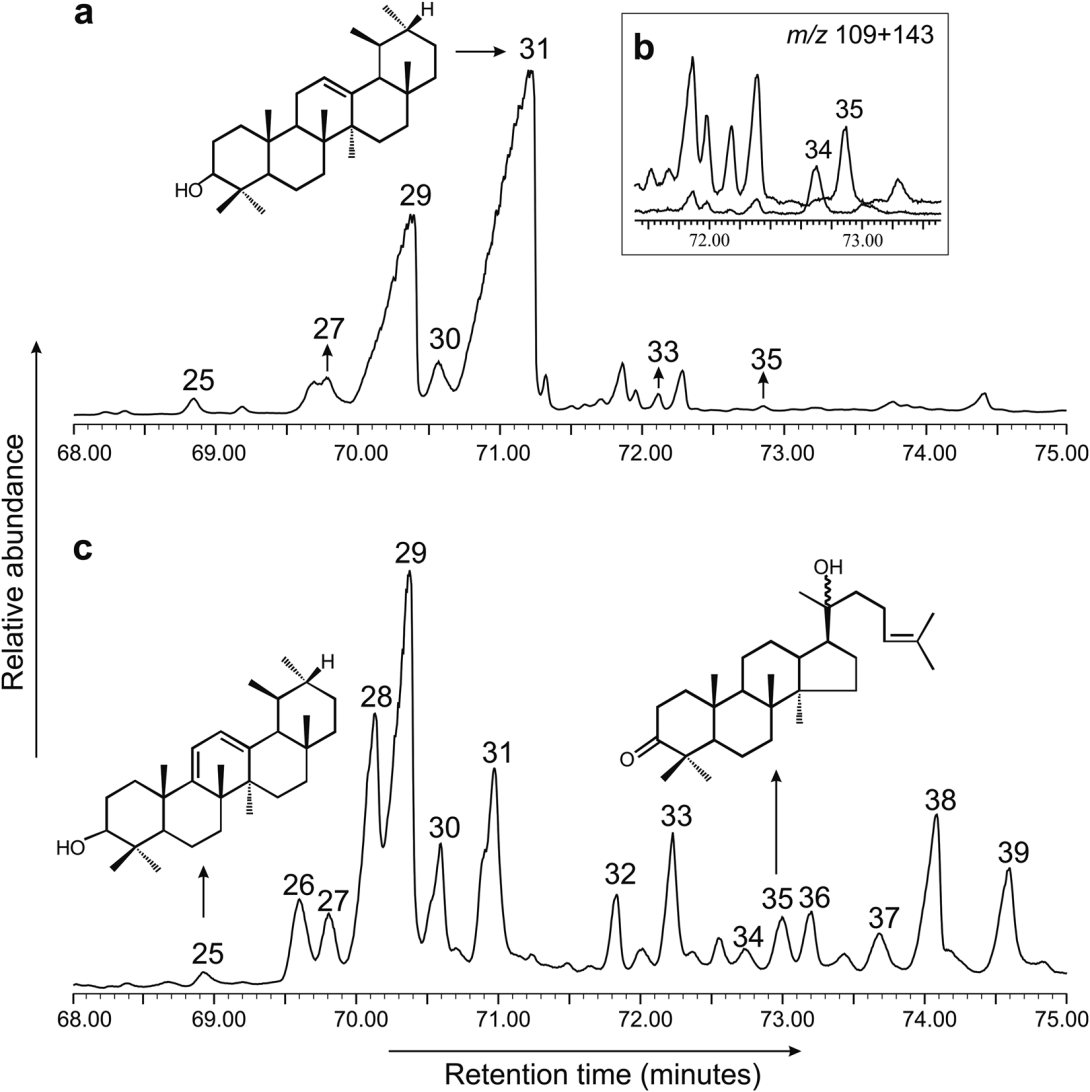
Gas chromatography-mass spectrometry traces (reconstructed) of (**a**) total ion current of the total extract of Miocene amber from Bhuban Formation, Assam Basin, northeastern India, (**b**) selected ion monitoring at *m/z* 109 + 143 of the total extract of Miocene amber from Bhuban Formation, Assam Basin, northeastern India, (**c**) total ion current of the total extract of the of the extant dammar resin of *Shorea robusta*. The numbers indicate triterpenoids present in the studied samples: 25, ursa-9(11),12-diene-3-ol; 26, dammarane based compound; 27, β-amyrone; 28, unknown triterpenoid; 29, β-amyrin, 30, α-amyrone; 31, α-amyrin; 32,lupenone; 33, hop-22(29)-en-3β-ol; 34, 20,24-epoxy-25-hydroxy dammaran-3-one; 35, hydroxydammarenone; 36, oleanoic aldehyde; 37, oleanolic aldehyde; 38, ursonic aldehyde; 39, ursolic aldehyde.

Gas chromatography-mass spectrometry analysis of the total extract of fossil resins includes both sesquiterpenoids and triterpenoids (Figs 1 and 2). Triterpenoids are more abundant than sesquiterpenoids. The major sesquiterpenoids include δ-elemene, α-copaene, β-caryophyllene, α-humulene, germacrene D, γ-cadinene, δ-cadinene, β–elemene and spathunenol. These volatiles are also abundantly found from the solvent extract of extant dammar resin (Fig 1b). In addition of bioterpenoids, aromatic geoterpenoids such as dihyro-*ar*-curcumene, calamenene and cadalene are detected in the total extract of the amber. α-Amyrin is the most abundant triterpenoid in the total extract of the amber (Fig 2a). The presence of oxygenated triterpenoids like α-amyrin and β-amyrin clearly suggests that the amber was derived from angiosperm trees^7^. We also detected dammarane-type triterpenoids such as hydroxydammarenone (20-hydroxy-24-dammaren-3-one) and 20,24-epoxy-25-hydroxydammaran-3-one.

The modern genera of higher plants secrete resin and use terpene-base defence mechanisms to repel insect pests and their fungal pathogens^8^. We have identified β-caryophyllene in the total extract of the Miocene amber in considerable amount. β-Caryophyllene is found in floral fragrance in more than 50% of angiosperm tree families^9^. This compound plays key roles in attracting pollinators and plant defence^9, 10^. It inhibits the growth and survival of insects feeding on resin-producing tree *Hymenaea* ^11^. This sesquiterpene attracts parasitic wasps to lay egg on lepidopteran larvae feeding on leaves^12^. Laboratory experiments show that β-caryophyllene serves as a defence against bacterial pathogens^9^. The volatile signalling used in plant defence occurs not only above ground, but also in the roots below ground. It has been demonstrated that the maize roots emit β-caryophyllene to attract enemies of root-feeding herbivore larve^13^. Germacrene D is an important sesquiterpene in the volatile component of the extant dammar resin^14^. We have identified this compound in the Miocene amber as well. It has been observed that germacrene D may have deterrent effects against herbivores and it has also been reported to have repellent activity against mosquitoes and aphids^15, 16^. Similarly, it has also been found that α-copaene is associated with fungal infections in crops^17^. Recent studies show that compounds like δ-elemene, β-elemene, α-caryophyllene are detected in the volatile fraction of *Eucalyptus* leaves attacked by the gall wasp^18^. Therefore, it is likely that these volatiles participate in plant defence mechanisms. In addition of sesquiterpenoids, 1,8 cineole and an unknown monoterpenoid occur in low abundance in the total extract of the studied amber. 1,8 Cineole is a toxin to marsupials^19^. It is worthwhile to mention that a few unaltered monoterpenoids was reported from Baltic amber and some Tertiary resins of Poland^20, 21^.

**Figure 3:**
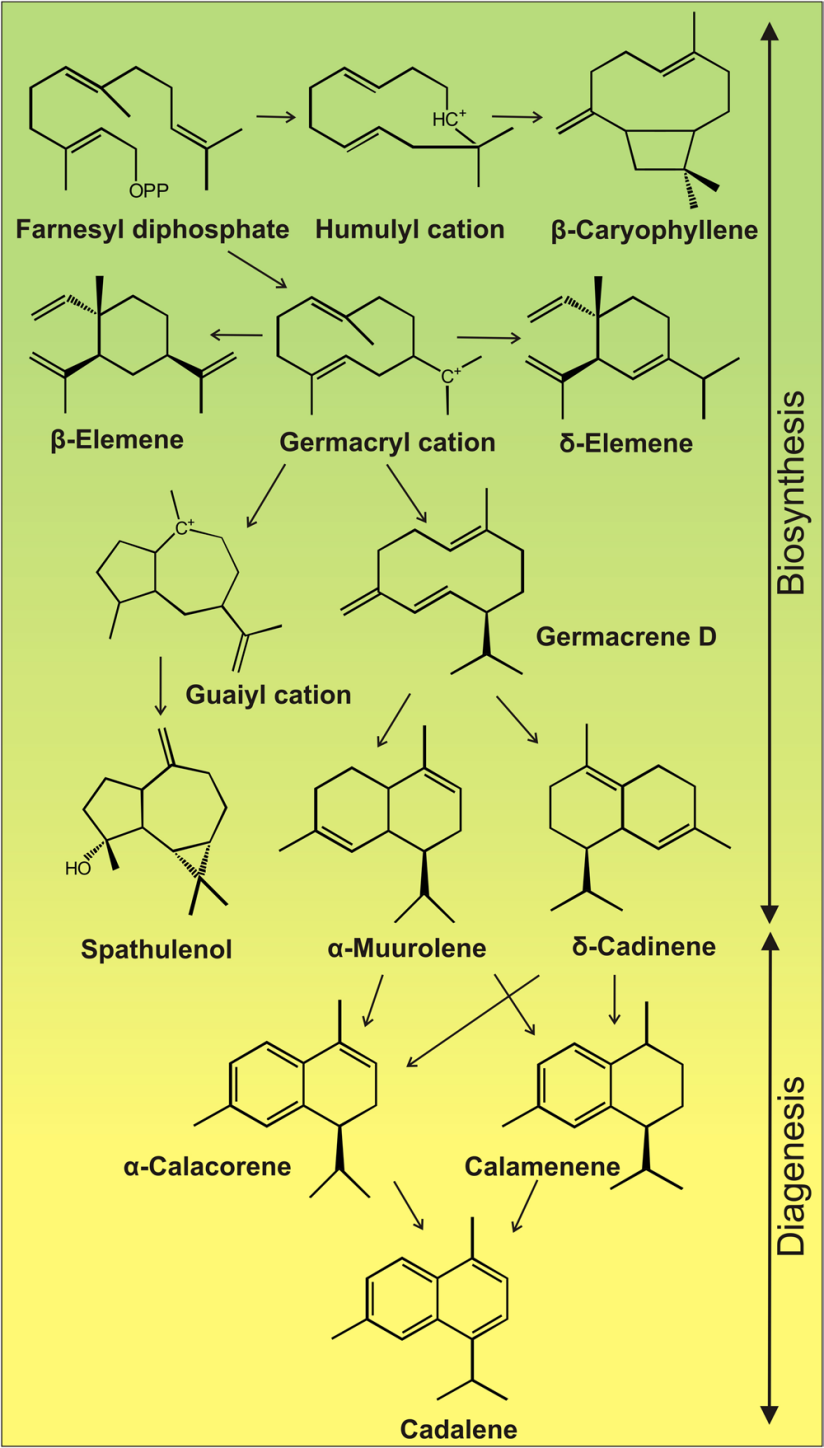
A scheme of biosynthesis and diagenesis of sesquiterpenoids, detected in the total extract of the Miocene amber from Bhuban Formation, Assam Basin, northeastern India.

The aromatic sesquiterpenoids such as dihydro-*ar*-curcumene, calamenene and cadalene are diagenetic products of bioterpenoids such as germacrene D and cadinenes^14^. Cadalene is produced due to dehydrogenation of cadinenes. Under acidic conditions germacrene D can be isomerized to cadalene-based structure^22^. The occurrence of these aromatic sesquiterpenoids in the studied amber suggests that the amber underwent mild diagenetic alterations (Fig 3).

Here we have demonstrated that volatile biomolecules may survive millions of years in sediments where suitable palaeoenvironmental conditions prevailed. To date, all the exceptional preservations of biomolecules were reported from organic-rich fine-grained sedimentary rocks^23, 24^. It is worthwhile to emphasize that we have recovered the studied amber blebs from an intraformational conglomerate horizon (Supplementary Figures 1, 2). We believe that the resinous material facilitates the preservation of volatile compounds, preventing oxidation and degradation; thus provides an exceptional taphonomic condition conducive to the preservation of the bioterpenoids. The Rock-Eval T_max_ of the fossil resin is 380 °C (Supplementary Table 1) which indicates that the amber underwent mild thermal alteration. It is very likely that low thermal maturity has an important role for the embalmment of these volatile organic molecules. Ambers facilitate morphological preservation of body fossils in deep time. The present study demonstrates that fossilized resins play a crucial role in the preservation of volatile biomolecules in the geosphere. There are several reports on organic geochemistry of Cenozoic fossil dammar resin^14, 25–27^. However, unaltered sesquiterpenoids were not reported in those studies.

The presence of dammarane compounds (e.g., hydroxydammarenone and 20,24-epoxy-25-hydroxydammaran-3-one) in the total extract of the amber clearly suggests that the fossil resins were produced by angiosperm tree family Dipterocarpaceae^28, 29^. 20,24-epoxy-25-hydroxydammaran-3-one is a diagenetic product of hydroxydammarenone. The aldehyde derivatives of α- and β-amyrin are observed in the extant dammar resin. However, these triterpenoids are absent in the Miocene amber. This suggests that aldehydes are readily susceptible to mild diagenetic alteration.

Today, Dipterocarpaceae contributes to 30% of the total area in the lowland evergreen forest of Southeast Asia^30^ and play an active role in the tropical rain forest ecology^31^. The earliest fossil record of dipterocarps comes from the early Eocene sediments of western India^32^. In south-eastern Asia, the fossil records of Dipterocarpaceae are found from middle Eocene onwards^27, 33^. Molecular phylogenetic studies suggest that this angiosperm family had a Gondwanan origin and migrated into Asia after the establishment of land connection between Indian and Asian plates^32, 34, 35^. The megafossil remains of Dipterocarpaceae have been reported from Miocene sediments of northeastern India^36^. Recently, fossil leaves resembling *Bauhinia* of the Fabaceae and *Holigarna* of the Anacardiaceae have been reported from these ambers^37^. Their presence and the present study indicate the existence of evergreen forests in the studied region during the Miocene.

Sesquiterpenes are synthesized via the isopentenyl pyrophosphate (IPP) intermediate following the mevalonate pathway^1^. The present study unequivocally demonstrates that the dipterocarps had evolved this biosynthetic mechanisms way back to early Miocene to produce these volatile plant metabolites. We postulate that these terpenoids were used by this angiosperm family as protective agents. Furthermore, the presence of unaltered biomolecules in the Miocene amber does suggest that the angiosperm family was using complex terpenoids in a manner similar to that seen in modern species of the family. This study offers a new approach to unravel the geological evolution of chemically-mediated interactions between plants and their immediate biotic environment.

## Material and Methods

### Amber Samples

Two amber blebs were recovered from the Thingdawl Hmar Veng quarry (23°45.184′N: 92°40.792′E), Mizoram northeastern India (Fig. S1). The sedimentary sequence is referred to as the Bhuban Formation, Assam Basin. Jauhri *et al*.^38^, on the basis of foraminifers, assigned the age of Upper Bhuban Formation as Burdigalian to Langhian (i.e from 20 Ma to 14 Ma). Palynological data suggest that the sediments of the Bhuban Formation were deposited in a near shore environment^39^. The collected amber specimens were extracted with a mixture of dichloromethane: methanol (9:1, v/v) by ultrasonication for 20 minutes. The total extracts were analyzed by gas chromatography–mass spectrometry. We also performed Rock-Eval pyrolysis of the amber to obtain thermal maturity of the studied sample.

### Gas Chromatography–Mass Spectrometry

The total resin extracts were analyzed by GC–MS with an Agilent 7890 A gas chromatograph attached to an Agilent 5975 C mass selective detector (MSD). A HP–5MS fused silica capillary column (30 m × 0.25 mm i.d., 0.25 µm film thickness) was used for the present study. Helium (He) was used as the carrier gas. The flow rate of He was 1 ml/min. The initial GC oven temperature was held at 40 °C for 5 minutes, subsequently increased to 310 °C at 4 °C/min, which was maintained for 5.5 minutes. Full scan analyses were performed over a mass range of 50–600 Da. The ion source of the MSD was operated in the electron ionization mode at 70 eV with a source temperature of 300 °C. We have identified the terpenoids with the help of authentic standards, mass spectra and elution pattern. Authentic standards such as 1,8 cineole, β-caryophyllene and α-humulene were procured from Sigma-Aldrich.

### Rock-Eval pyrolysis

A Rock Eval 6, manufactured by Vinci Technologies® was used for the present study. We have pyrolyzed 3 mg of amber sample. Initially the sample was pyrolyzed in an inert atmosphere of nitrogen in the pyrolysis oven. The initial temperature of the pyrolysis oven was maintained at 300 °C for 5 min. Subsequently, this temperature was raised to 650 °C at a rate of 25 °C/min. Hydrocarbons released from the amber are measured by flame ionization detection (FID). The oxidation phase was carried out in the oxidation oven. The temperature began with 300 °C, held for 5 min. Then it was increased to 750 °C at the rate of 25 °C/min. The released CO and CO_2_ were detected by online infrared detector.

## Electronic supplementary material

Supplimentary file 1
