# Supplementary material for: Remarkable preservation of terpenoids and record of volatile signalling in plant-animal interactions from Miocene amber
Source: Sci Rep. 2017 Sep 8;7:10940. doi: 10.1038/s41598-017-09385-w (PMC5591258; doi:10.1038/s41598-017-09385-w)
Supplement: Supplementary file 1 — Supplimentary file 1 [file 41598_2017_9385_MOESM1_ESM.pdf]

Supplementary Materials:

Remarkable preservation of terpenoids and the record of volatile signaling in plant-animal interactions from Miocene amber

Suryendu Dutta[1*], Rakesh C. Mehrotra[2], Swagata Paul[1], R. P. Tiwari[3,†], Sharmila Bhattacharya[1], Gaurav Srivastava[2], V.Z. Ralte[3,] , C. Zoramthara[3]

[1]Department of Earth Sciences, Indian Institute of Technology Bombay, Mumbai-400076, India
[2]Birbal Sahni Institute of Paleosciences, Lucknow 226007, India
[3]Department of Geology, Mizoram University, Aizawl 796004, India
†Present address: Dr. H. S. Gour Vishwavidyalaya, Sagar 470003, Madhya Pradesh, India
†† V.Z. Ralte deceased 19th August, 2015

Fossil locality:

The fossil locality, Thingdawl Hmar Veng quarry (23 45.184 N; 92 40.792 E), falls in the Upper Bhuban Unit of the Bhuban Formation of Surma Group (Karunakaran 1974; Ganju 1975). It is located about 75 km from Aizawl city on the right side of the National Highway No. 54. The lithocolumn exposed in this quarry is provided in figure S1b. There are two fossiliferous beds in this locality: the lower one consists of about 5 m thick bluish grey silty-sandstone, while the upper is made up of about 1 m thick buff sandstone. The lower unit is fine grained, moderately hard and compact and flaser beddings and ripple-drift cross laminations are the dominant sedimentary structures in it. The fossils found in this bed are poorly preserved and rocks are too hard to extract them in good form. The intraformational conglomeratic layer lying immediately above the silty-sandstone bed is somewhat calcareous at places containing the amber fossil.

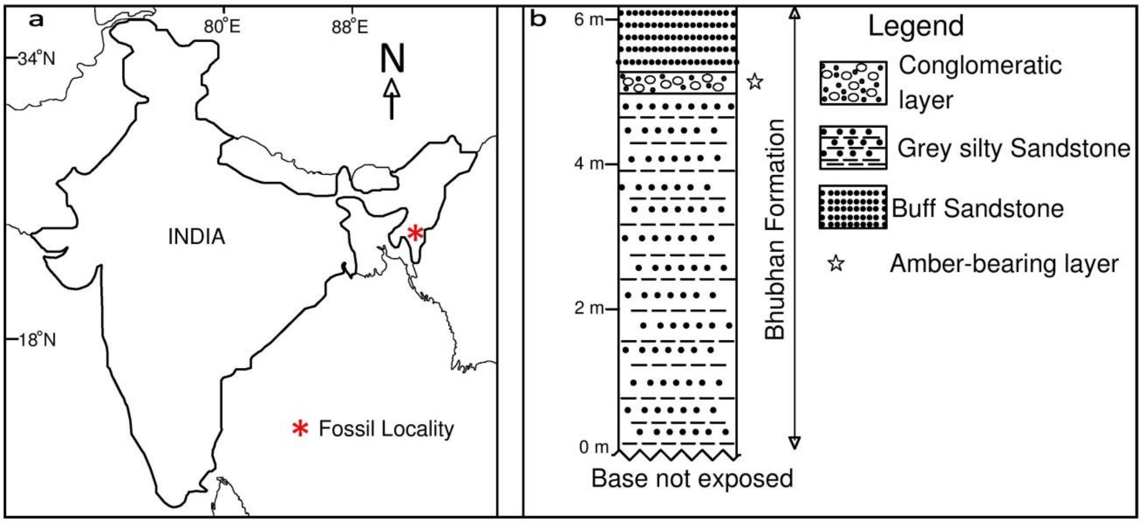

Figure S1: (a) Map showing location of amber-bearing sedimentary deposits and (b) litholog of the sedimentary sequence (figure was prepared using AutoCAD 2015, https://www.autodesk.in/).

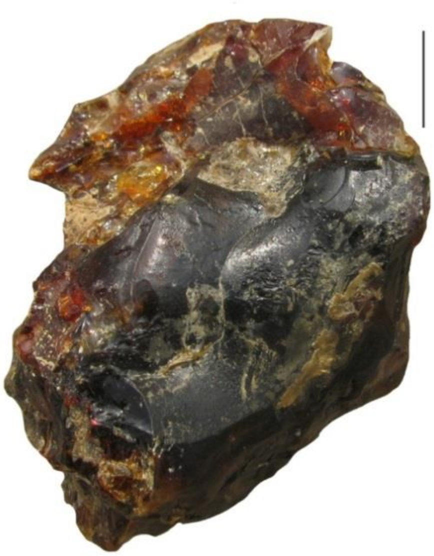

Figure S2: Photograph of the Miocene amber from Bhuban Formation, Assam Basin, northeastern India (scale= 1 cm).

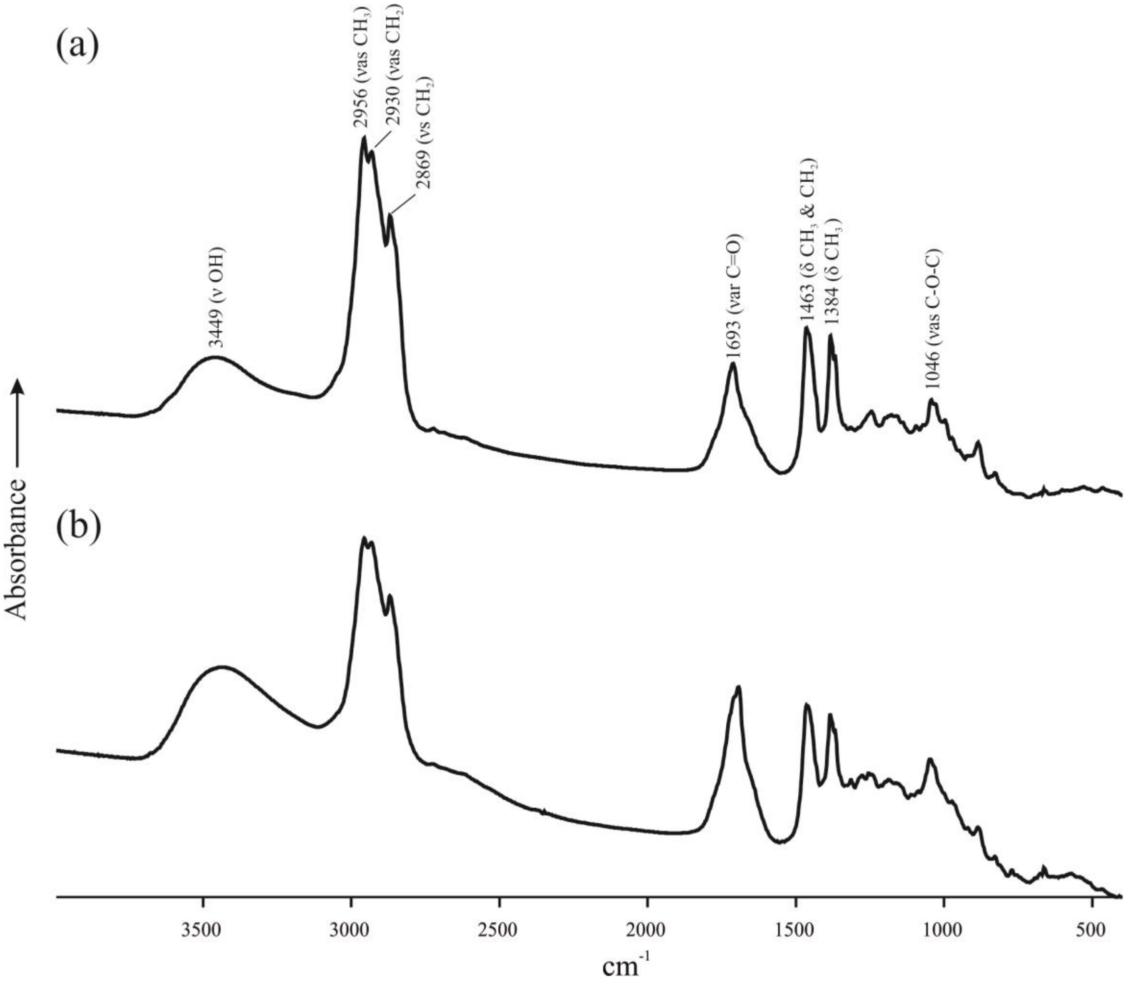

Figure S3: FTIR spectra of (a) Miocene amber from Bhuban Formation, Assam Basin and (b) extant dammar resin collected from *Shorea robusta* (family Dipterocarpaceae). Assignments of absorption bands and mode of vibration (δ= deformation; ν= stretching; s = symmetric, as = asymmetric) are given in parentheses.

Fourier Transform Infrared Spectroscopy (FTIR):

Methods:

FTIR spectroscopic analyses from 4000 to 450 cm^{-1} were carried out in transmission mode using Vertex 80 FTIR spectrometer manufactured by Bruker. Data were processed using opus

software. We used powder resin samples and prepared the KBR pellets. Spectra were obtained for a defined area by accumulating up to 512 scans with a spectral resolution of 4 cm^{-1}.

Results:

FTIR spectra of Miocene amber and extant dammer resin are shown in Figure S3. Prominent peaks in the range of 3000-2800 cm^{-1} can be assigned as aliphatic CH_x stretching vibrations. Strong narrow absorptions centered in the range from 2960–2950 cm^{-1} and from 2870–2850 cm^{-1} are due to asymmetric stretching vibration from CH_3 and symmetric stretching vibrations from CH_2 groups, respectively. A peak a 2930 cm^{-1} is due to asymmetric stretching vibration from CH_2 .A prominent peak of aliphatic CH_x deformation at 1463 cm^{-1} indicates high CH_2 abundances. The abundant absorption at 1693 cm^{-1} is attributed to aromatic carbonyl/carboxyl C=O groups. FTIR data clearly reveal that the chemical composition of Miocene amber is similar with that of extant dammar resin derived from dipterocarps. Previous studies on fossil dammar resin using FTIR spectroscopy show similar geochemical characteristics (e.g. Mallick et al., 2009).

Table S1: Rock-Eval pyrolysis data of Miocene amber from Bhuban Formation, Assam Basin, Northeastern India.

TOC (%)	T_{max} (°C)	S_1 (mgHC/ g rock)	S_2 (mgHC/ g rock)	S_3 ($mgCO_2$/ g rock)	PI	HI (mgHC/ gTOC)	OI ($mgCO_2$/ gTOC)
53.56	380	103.01	420.86	28.29	0.2	786	53

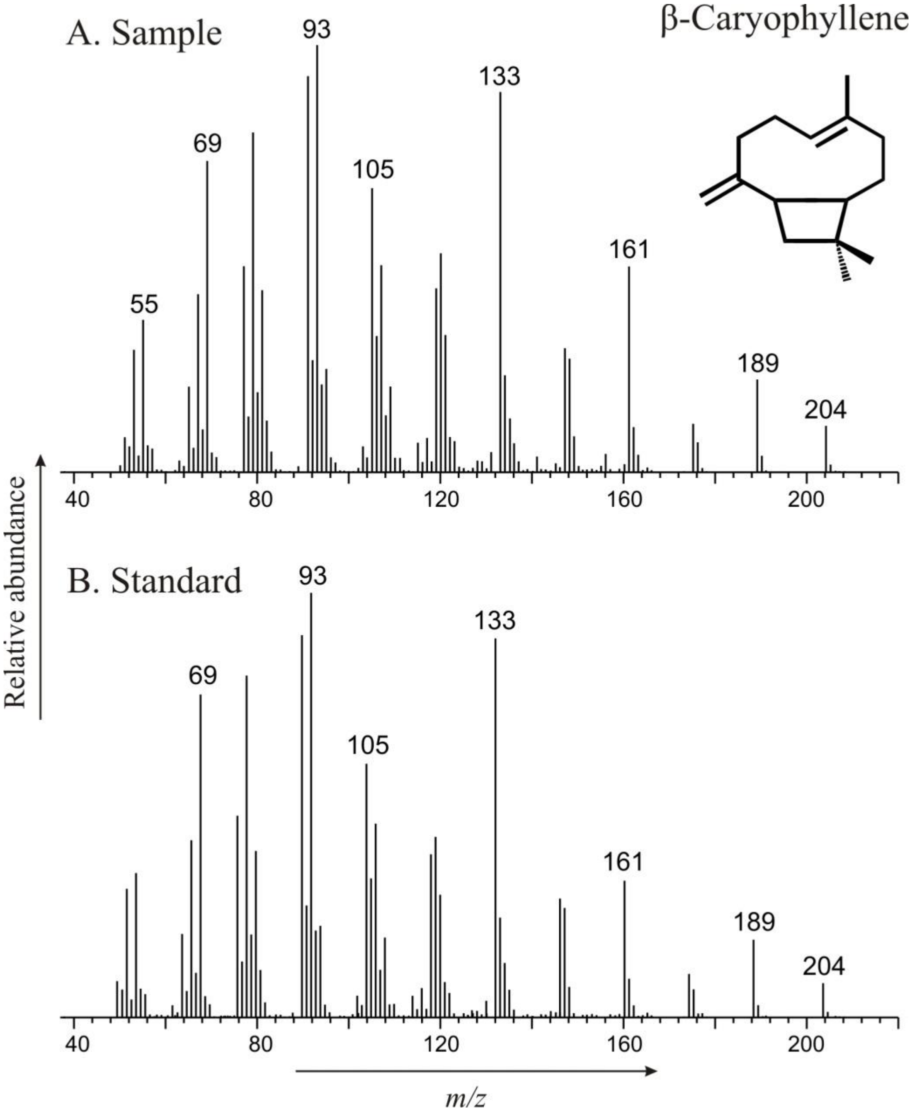

Figure S3: Mass spectra of β-caryophyllene

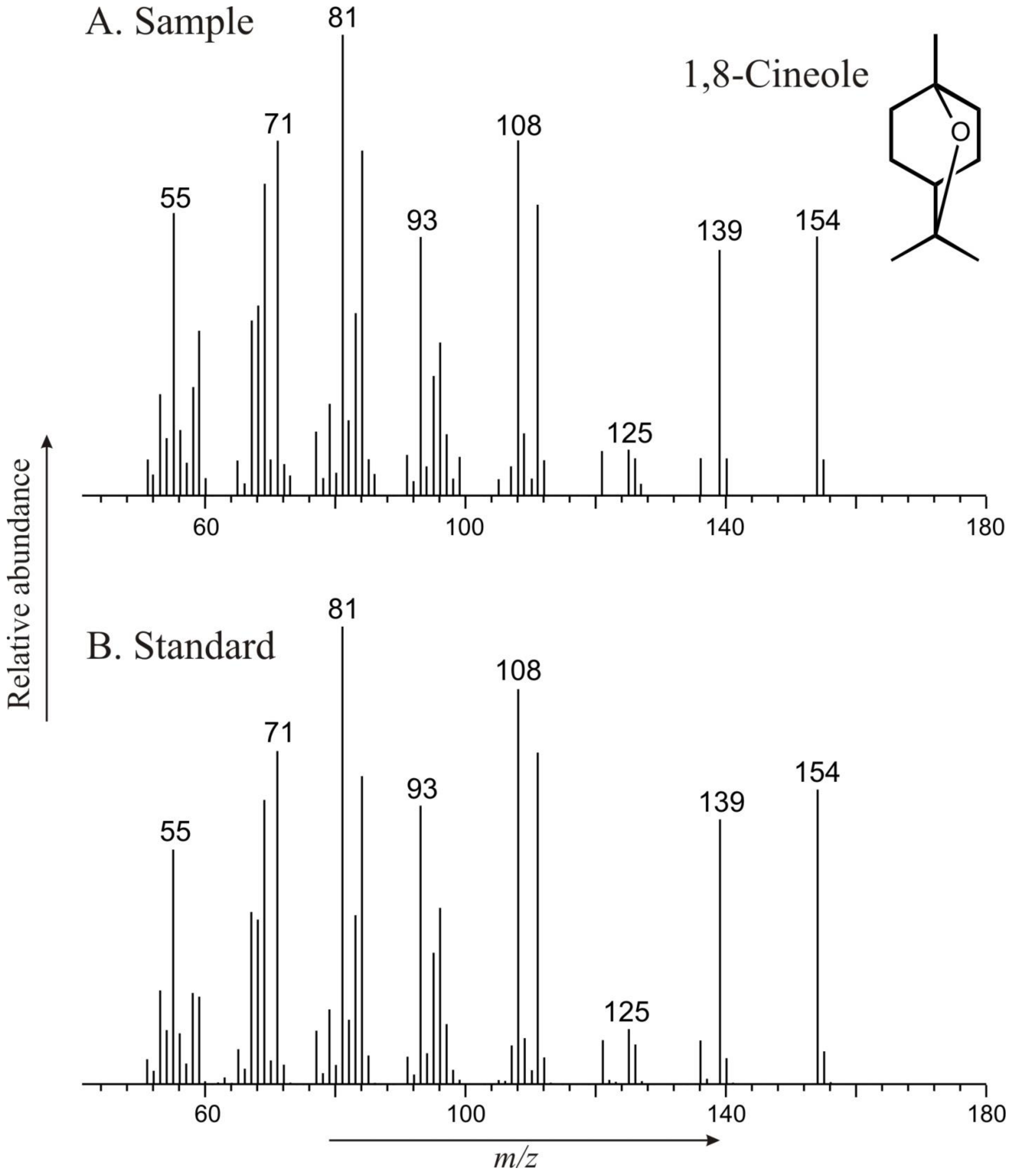

Figure S4: Mass spectra of 1,8-cineole

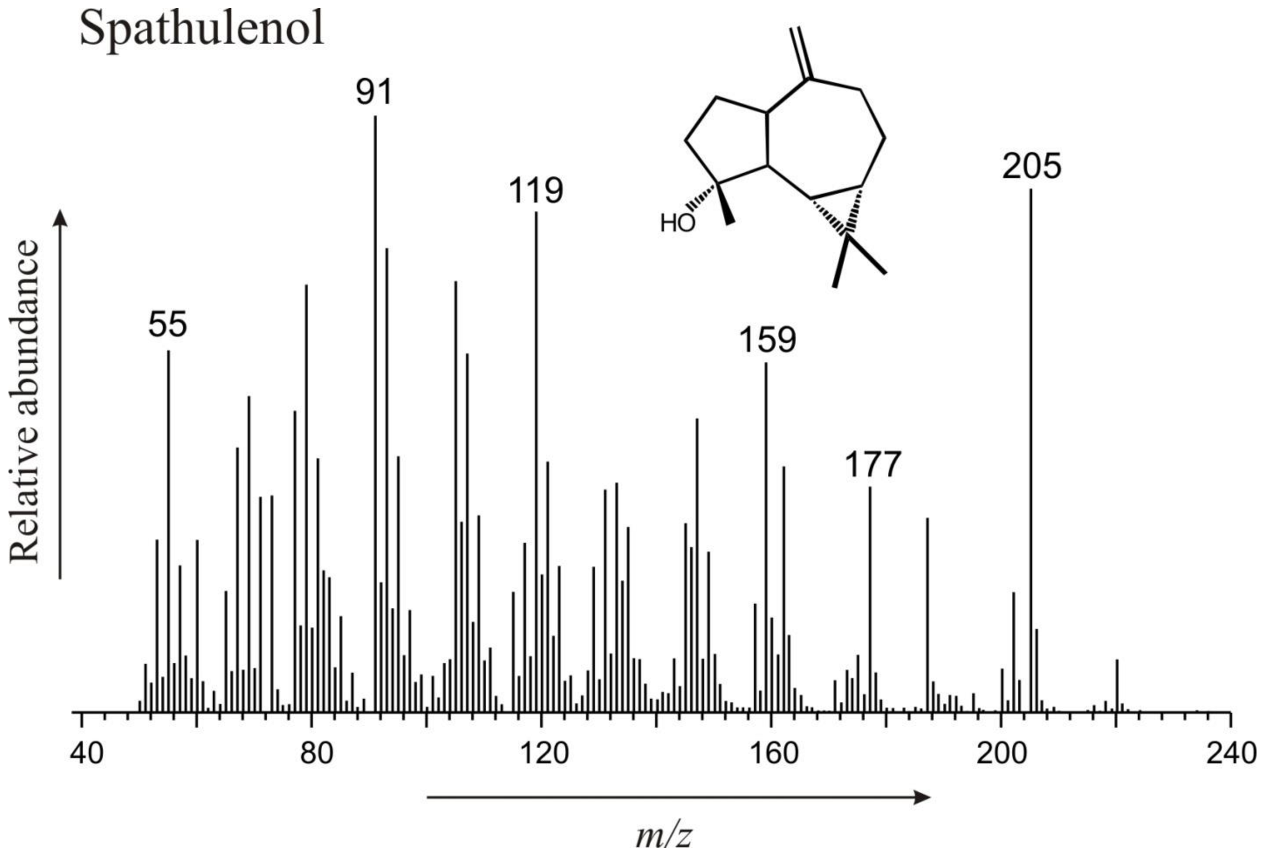

Figure S5: Mass spectra of spathulenol

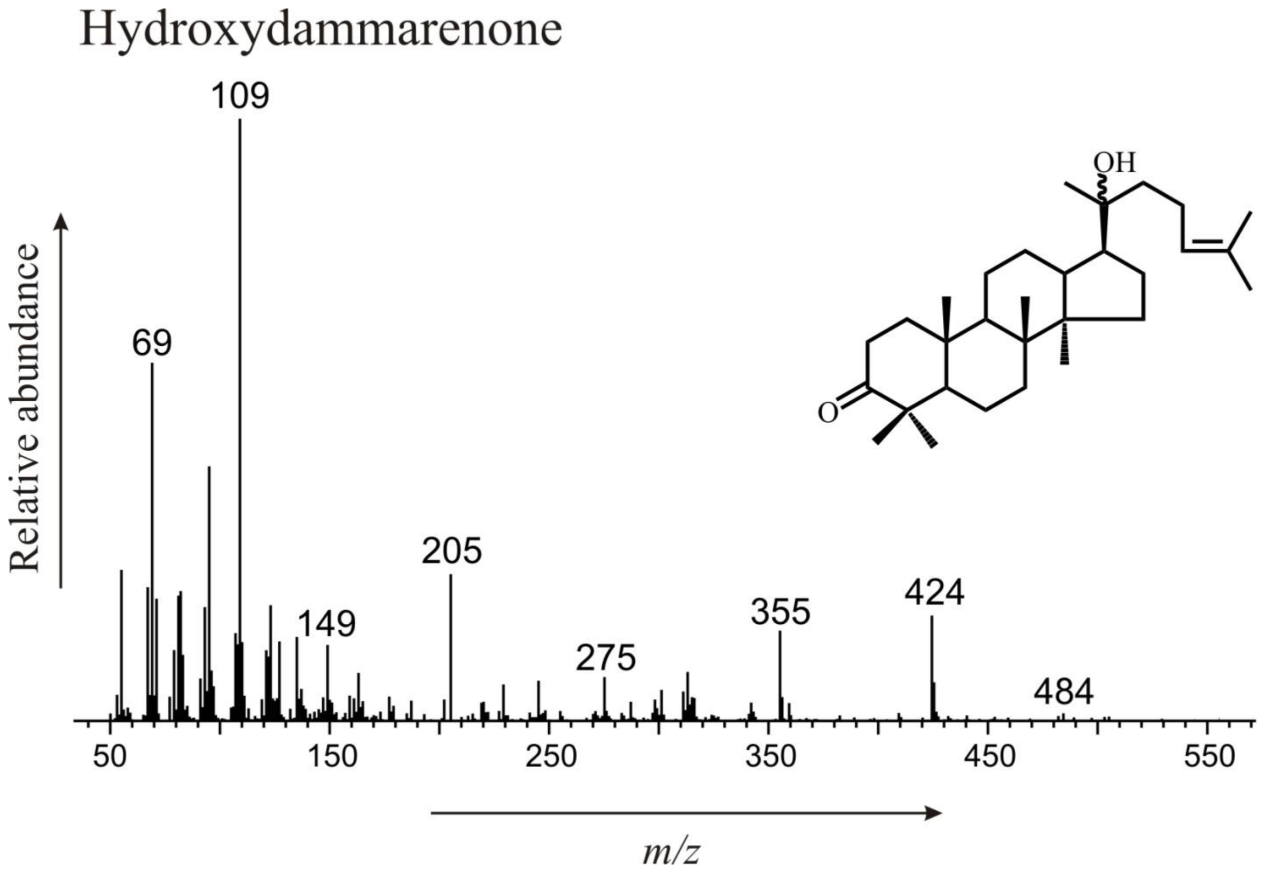

Figure S5: Mass spectra of hydroxydammarenone

References

Karunakaran, C. Geology and mineral resources of the states of India. Part IV Arunachal Pradesh, Assam, Manipur, Meghalaya, Mizoram, Nagaland and Tripura. *Geological Survey of India Miscellaneous Publication,* **30**, 1–124 (1979).

Ganju, J.J. Geology of Mizoram. *Bulletin of Geological Mining & Metallurgical Society of India*, **48**, 17–26 (1975).
